# Temperature Changes in the Pulp Chamber During Orthodontic Bonding Using a High-Power Light-Emitting Diode Device With Two Different Exposure Times and Intensities: An In-Vitro Study

**DOI:** 10.7759/cureus.61287

**Published:** 2024-05-29

**Authors:** Alia Alsafadi, Nada Rajeh, Mohammad Y Hajeer, Mowaffak A Ajaj, Ahmad S. Burhan

**Affiliations:** 1 Department of Orthodontics, Faculty of Dentistry, University of Damascus, Damascus, SYR

**Keywords:** composite curing, bonding orthodontic brackets, cooling time, led device for orthodontic curing, exposure time, high power led, thermal imaging camera, variation in temperature, pulp chamber

## Abstract

Objective: This study used a high-power light-emitting diode (LED) device to evaluate the effects of two exposure times and intensities on pulp chamber temperature and cooling time during bracket bonding.

Materials and methods: Sixty upper premolars were used in the sample in this study. These premolars were split into two main groups based on the exposure time and intensity: the first group employed a traditional curing mode (TCG) for 20 seconds with an intensity of 1200 mw/cm^2^, whereas the second group had a quick curing mode (QCG) for 3 seconds with an intensity of 2500 mw/cm^2^. The pulp chamber's temperature variations and cooling times were recorded using a thermal imaging camera. The Mann-Whitney U test was used to find differences between the two-group comparison of the pulp chamber's temperature and cooling time.

Results: The two groups had statistically significant differences regarding the temperature increase in the pulp chamber and cooling time (p > 0.001). The mean temperature increase in the traditional curing group was 3.52°C, which is greater than that in the quick curing group (i.e., a mean value of 1.28°C). The mean cooling time in the traditional curing group was 38.83 seconds, which is greater than that in the quick curing group (9.97 seconds).

Conclusions: Reducing the exposure time to 3 seconds and increasing the intensity to 2500 mw/cm^2^ is considered safer for the pulp chamber during and after the curing process.

## Introduction

Light-emitting diode (LED) light-curing units (LCUs) are now widely used in clinical dentistry. The popularity of these devices is due to their efficiency and design as they are battery-powered and frequently do not require cooling fans like conventional LCUs [1]. Over a decade ago, highly powerful LED LCUs were commercially accessible, promoting improved polymerization of resin-based materials with reduced exposure times [2]. These devices may produce light with an intensity of more than 2000 mw/cm^2^, and some manufacturers support exposure durations of no more than 5 seconds (sec). As a result, these high-power LED LCUs produce heat in the pulp chamber [3].

Dental professionals have long been worried about the potential harm caused by temperature increases on pulp tissue during restorative treatments. Cavity preparation, polymerization of lining, restorative materials, and orthodontic bonding brackets can cause a temperature increase at the cavity floor, potentially leading to an increase in intra-pulpal temperature [4]. Zach's research on monkey teeth found that a 5.5°C increase in pulp temperature produced significant damage, causing the loss of vitality in 15% of the teeth [5].

The exothermic polymerization of the material and the heat output from dental light-curing devices are related to the increase in temperature that occurs during the curing of light-activated restoratives. This expansion occurs with increasing radiation time and decreasing material thickness [6]. Few studies evaluated the heat increase in the pulp chamber caused by different exposure times and intensity levels when comparing the plasma arc light with LED light [7]. Additionally, a comparison of halogen light and LED light with the aid of a thermal camera during orthodontic bonding was made [8,9]. Many studies have evaluated the thermal effects of different curing lights using thermocouple wires to measure the increase in intra-pulpal temperature [8-10]. Several studies have examined the effects of using LED-curing lights to shorten the exposure time, which contributes to increasing its intensity [3,11,12]. Consider the fact that two crucial variables affecting one another throughout this process are time and intensity [13]. Additionally, during the composite resin polymerization process for various LCUs, an elevated intra-pulpal temperature has been shown in the literature [14,15].

The current study's objective was to determine how exposure time and intensity affected the pulp chamber's temperature during orthodontic bonding using a thermal imaging camera when a high-power LED was used with two light intensities.

## Materials and methods

Study sample

**Figure 1:**
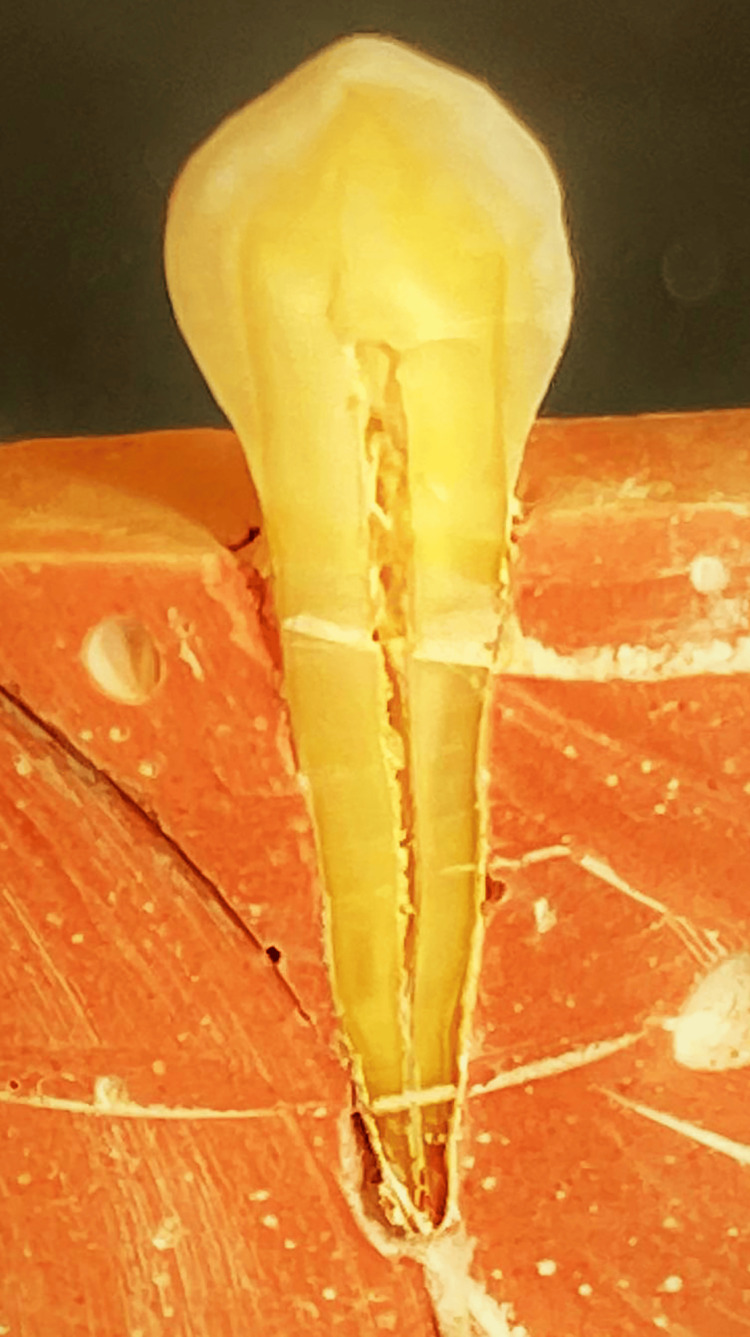
A cross-section of an examined premolar by splitting the tooth into the buccal and palatal halves

This in vitro study was carried out on 60 human upper premolar teeth without caries or fillings and no obvious buccal defects that had been extracted from adults for orthodontic reasons. After extraction, all teeth were handled following the technical specification ISO/TS 11405:2003. The teeth were cleaned to remove the blood and any remaining periodontal tissue; the teeth were immersed in 10% formalin for 24 hours. They were then divided into two halves: buccal and palatal, with the buccal halves kept in distilled water at 4°C (Figure 1).

For bonding, buccal surfaces of all premolar teeth were etched for 30 sec using a 37% phosphoric acid gel, followed by a 30-second water wash and thorough drying. The etched enamel was covered with a layer of bond, and the metal brackets (0.022 MBT; MiniMaster, American Orthodontics, Sheboygan, WI) were covered with resin cement (Resilience, Ortho Technology, Florida). The composite was cured using an LED (Guilin Woodpecker Medical Instrument Co, China) with an intensity of 1200 mw/cm² to ~2500 mw/cm² and 390-480 nm. For every specimen, the light tip's distance from the brackets was maintained at a closed distance.

Teeth with bonded brackets were divided into two groups (each with 30 teeth) according to the curing time and power intensity: the Traditional Curing Group (TCG), consisting of 30 upper premolars, which has a power intensity of 1200 mw/cm^2^ for 20 sec, and the Quick Curing Group (QG) consisting of 30 upper premolars, which has a power intensity of 2500 mw/cm^2^ for 3 sec.

Outcome assessment

**Figure 2:**
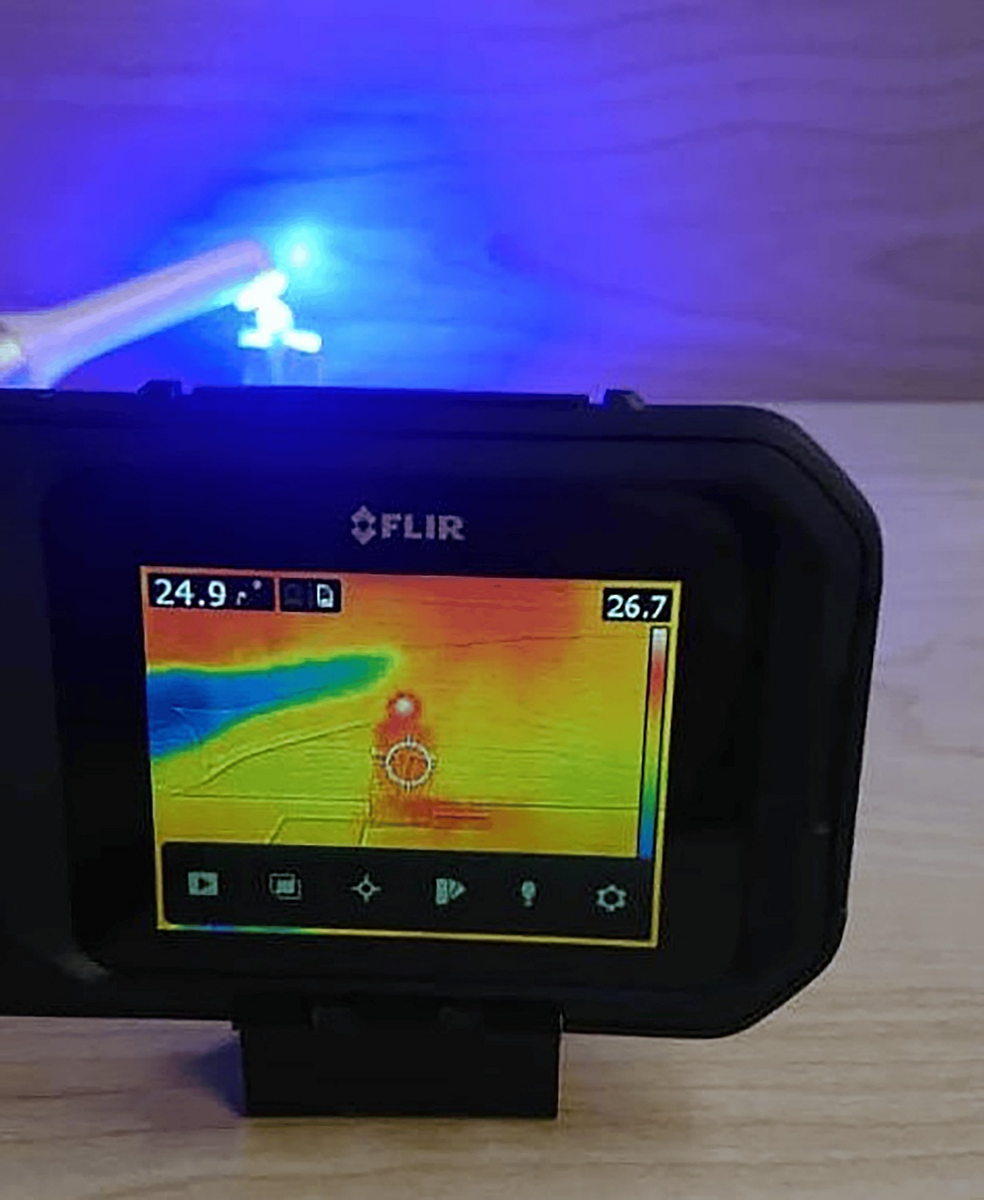
The thermal camera used in the current study when curing the composite material

The pulp chamber temperature was measured with a thermal camera at the Higher Institute of Laser and Applications, Damascus University, Damascus, Syria. The ambient temperature was adjusted to 26 ± 0.9°C before any thermal measurements were taken. The temperature variations during and after bonding were captured using a FLIR C2 thermal camera (FLIR Systems, Inc, PORTLAND, USA; Figure 2).

The samples were moved using plastic forceps to prevent any heat impacts for temperature measurements. Based on the recorded temperature of the LED equipment head, the specimens were positioned six inches away from the camera. Then, 2 sec before the commencement of curing, the recording was initiated, and the temperature variations in the pulp chamber during bonding were measured. For every specimen, the cooling time was noted, which is the time taken to cool down from the greatest temperature value to the beginning degree.

Statistical analysis

SPSS version 20 (IBM Corp., Armonk, NY) was used for the statistical analysis. The Mann-Whitney U test was employed for comparisons between the two groups, and the significance level was set at 0.05.

## Results

**Figure 3:**
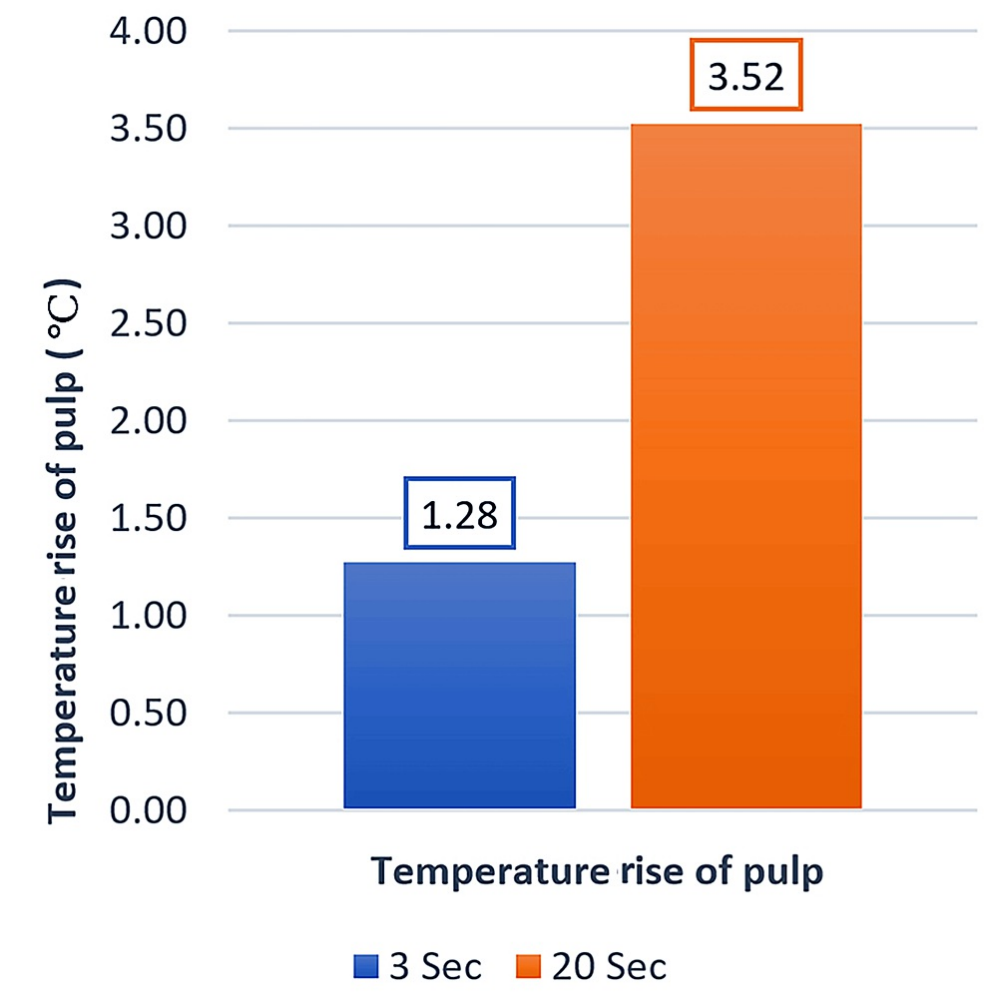
Average temperature increase of the pulp chamber in the two study groups

**Figure 4:**
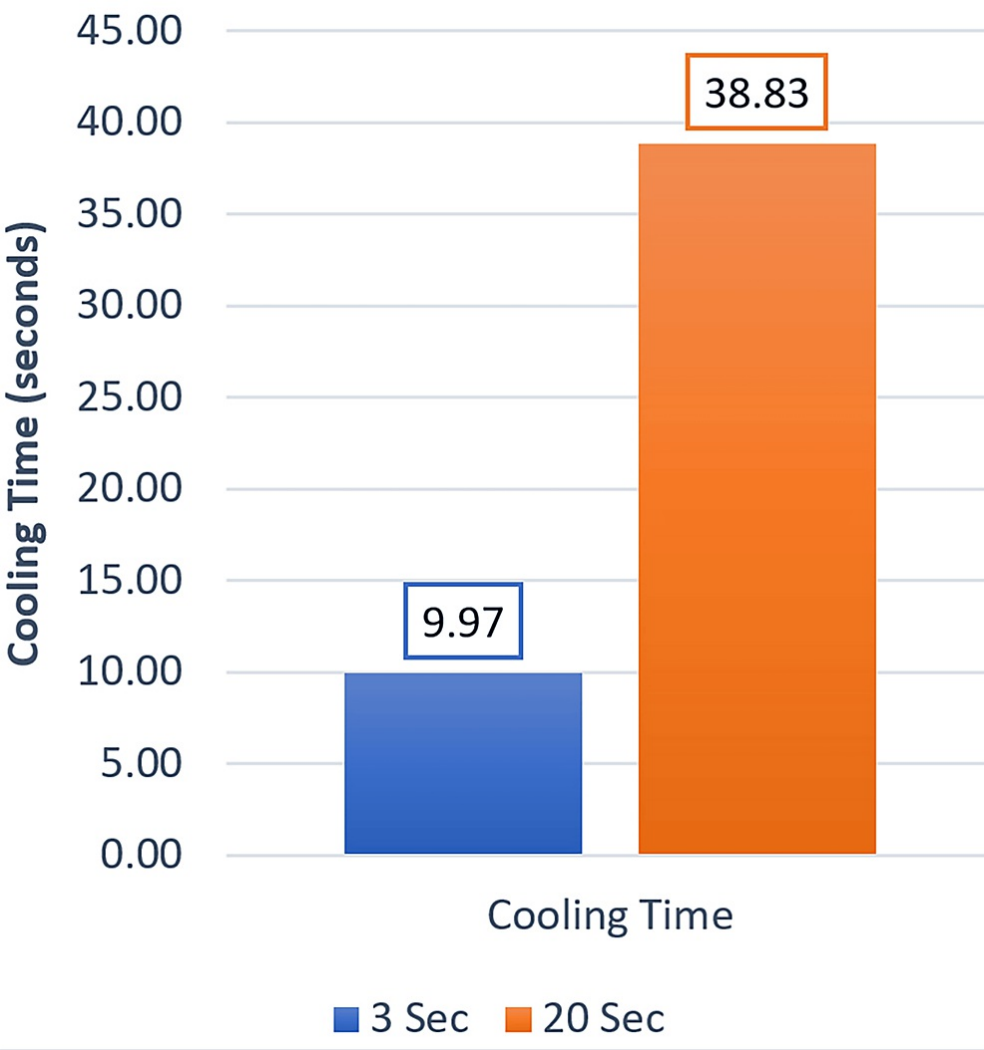
Average cooling time in the two study groups

The two groups had statistically significant differences regarding the temperature increase in the pulp chamber and cooling time (p > 0.001). The mean temperature increase in the TCG was 3.52°C, which is greater than that of the QCG (i.e., 1.28°C; Figure 3). The mean cooling time in the TCG was 38.83 sec, which was greater than that of the QCG (i.e., 9.97 sec; Figure 4).

The TCG with a power intensity of 1200 mw/cm^2^ for 20 sec recorded a higher temperature increase in pulp than the QCG, as it was 4.5°C in TCG with a mean cooling time of 58 sec, whereas the QCG with a power intensity of 2500 mw/cm^2^ for 3 sec had a mean temperature increase of 2.9°C and mean cooling time of 20 sec (Table 1).

**Table 1:** Descriptive statistics of temperature increase of pulp and cooling time in the two groups and the results of significance testing ^a^ The traditional curing group used an intensity of 1200 mw/cm^2^, whereas the quick curing group used an intensity of 2500 mw/cm^2^. ^b^ Mann-Whitney U Test. * Significant at the 0.05 level. SD: Standard deviation.

Variables	Traditional curing group (20 sec)^a^			Quick curing group (3 sec)			Mean difference	95% confidence interval of the difference		P-value^b^
	Mean ± SD	Min	Max	Mean ± SD	Min	Max		Lower	Upper	
Temperature increase of pulp	3.52 ± 0.83	1.2	4.5	1.28 ± 0.61	0.5	2.9	-2.25	-2.62	-1.87	<0.001*
Cooling time	38.83 ± 9.31	25	58	9.97 ± 3.84	6	20	-28.87	-32.59	-25.15	<0.001*

The two groups had statistically significant differences in the temperature increase in the pulp chamber and cooling time. The temperature increase in the pulp chamber in TCG was greater by a mean difference of 2.25°C compared to the QCG, and the cooling time in TCG was greater by a mean difference of 28.87 sec compared to QCG.

## Discussion

Adequate polymerization is critical for achieving good physical properties and clinical performance of orthodontic composite bonding, while inadequate polymerization can lead to poor physical properties, oral solubility, increased microleakage, and bonding failure. Adequate polymerization can occasionally be linked to the power intensity of the light sources or the curing duration, particularly in LED-curing units. The temperature changes that occur during light curing are widely recognized, but the temperature changes in the pulpal chamber during curing application are not well-known [8].

An infrared camera was used to assess the increase in temperature of the pulpal chamber. This technique is more accurate than the thermal couple method because the measurement error increases when thermal couples are used because of inadequate contact between the probe and the tooth structure (such as an air gap).

In general, a temperature increase in the pulp chamber higher than 5.5°C was concluded to cause irreversible damage to the pulp tissue, relying on the findings of Zach [5]. Even if the pulpal reaction after heat generation in animal teeth cannot be transferred completely to human teeth, one important conclusion must be addressed: heat generation may damage pulpal tissue. To protect the adjacent teeth from potential heat-related damage during orthodontic bonding, the current study assessed the increase in intra-pulpal temperature when using high-power LEDs with two different exposure times and intensity levels in addition to cooling time. This could give us the ideal needed time before curing the adjacent tooth. Nonetheless, increased irradiation from high-energy output lights is thought to be a primary cause of pulp injury [8,11]. One of the most essential aspects of investigating the effects of LED exposure on pulp temperature is determining an exact link between light exitance, radiant exposure, and pulp temperature rise [16].

This is a rare study to examine how two various exposure times and intensities affect the pulp chamber's temperature during bracket bonding using a high-power LED. Based on the findings of this investigation, the TCG experienced a higher temperature increase in the pulp chamber during curing than the QCG. The traditional curing group's average temperature increase was 3.52°C, compared to the QG’s, which was 1.28°C. As a result, the intra-pulpal temperature increased as the exposure time extended. Additionally, none of the two study groups experienced a temperature increase above the threshold of 5.5°C, as reported by Zach [5]. The highest recorded temperature increase was 4.5°C in TG, which could be explained by the energy absorbed by the tooth from light radiation and the composite's polymerization process. In orthodontic research, light intensity plays a less important role than curing time because the energy emitted by the device will pass via the bracket's thickness, the adhesive material, and the tooth. This is nearly consistent with a recent study [17] that revealed that high-power LEDs produced the least temperature change when comparing halogen lights with LEDs of varying powers and exposure times.

According to the study's findings, there were statistically significant differences in the cooling times of the two study groups. It was discovered that the TCG (with an exposure time of 20 sec and an intensity of 1200 mw/cm^2^) took the longest for the pulp chamber to reach its starting temperature after curing (58 sec), while the QCG (with an exposure time of 3 sec and an intensity of 2500 mw/cm^2^) took 20 sec. Given that light energy is the product of exposure time and light intensity, this could result in the curing device reaching its maximum energy [17]. This is nearly in accordance with a recent study [7] that examined two distinct light sources, plasma arc light and LED light (VALO ortho cordless), with varying power and exposure times. The study discovered that when using a high-power LED (VALO), the longest exposure time was 10 sec, requiring 77.19 sec for cooling. The cooling time increased in parallel with the curing time, and it took a minute to return to the maximum primary temperature.

Limitation of the current study

Our experimental design did not account for heat conduction in the tooth due to blood circulation in the pulp chamber and flow in dentin tubules during the in-vivo orthodontic adhesive bonding procedure. In addition, the surrounding periodontal tissues can assist heat convection in vivo, reducing the increase in intra-pulpal temperature.

## Conclusions

Based on the findings of this investigation, the TCG with 20 sec of exposure experienced a higher temperature increase in the pulp chamber during curing than the QCG with 3 sec of exposure. The TCG's average temperature increase was 3.52 °C, compared to 1.28 °C in the QCG. The two study groups had statistically significant differences in the cooling times. The TCG required about one minute on average for the pulp chamber to reach its starting temperature, whereas the QCG required only 20 sec. For the safety of the pulp chamber both during and after the curing process, it is suggested that shortening the exposure time to 3 sec and increasing the curing intensity level to 2500 mw/cm^2^ is more efficient than the traditional curing method.
